# Is catheterization via distal transradial access feasible in children? From vessel diameter perspective

**DOI:** 10.3389/fcvm.2024.1428083

**Published:** 2024-08-02

**Authors:** Yidong Zhao, Tao Chen, Ling Yang, Wenjie Mao, Yu Wan, Liwen Zhang, Heng Ding, Gaojun Cai, Zhiying Huang

**Affiliations:** ^1^ Department of Pediatrics, The Second People’s Hospital of Changzhou, Affiliated Hospital of Nanjing Medical University, Changzhou, Jiangsu, China; ^2^ Department of Cardiology, Wujin Hospital Affiliated with Jiangsu University, The Wujin Clinical College of Xuzhou Medical University, Changzhou, Jiangsu, China; ^3^ Department of Respiratory and Critical Care Medicine, The Affiliated Changzhou Second People’s Hospital of Nanjing Medical University, Changzhou, China

**Keywords:** cardiac catheterization, children, congenital heart disease, radial artery, unusual access

## Abstract

**Background:**

Distal radial artery (DRA) access is an infrequent alternative access for pediatric catheterization. The feasibility of using the DRA for arterial catheterization in children depends on the vessel's size.

**Objectives:**

This study aims to provide a reference for pediatric catheterization via DRA access by evaluating the diameter of the DRA in the anatomic snuffbox (AS).

**Methods:**

We conducted a retrospective review of clinical and vascular ultrasound data of 412 children (ages 3–12) who were scheduled for arterial blood gas analysis via the DRA due to serious respiratory diseases between June 2023 and October 2023.

**Results:**

The corrected DRA diameter in the AS was 1.97 ± 0.37 mm overall, with no significant difference between males (1.98 ± 0.38 mm) and females (1.95 ± 0.35 mm) (*p *= 0.457). The anteroposterior, transverse, and corrected DRA diameters increased significantly with age (*p *< 0.05). The DRA diameter was significantly smaller than the proximal radial artery (PRA) diameter (1.97 ± 0.37 mm vs. 2.05 ± 0.33 mm, *p *< 0.001) but larger than the ulnar artery (UA) diameter (1.97 ± 0.37 mm vs. 1.88 ± 0.33 mm, *p *< 0.001). The proportions of patients with a DRA diameter greater than 2.0 mm and 1.5 mm were 38.83% and 86.89%, respectively. The proportions of patients with DRA diameters >2.0 mm and >1.5 mm increased significantly with age (*p* < 0.01). The percentages of individuals with a DRA/PRA ratio ≥1.0 were 55.10% overall, 52.12% in males, and 58.60% in females. DRA diameter showed significant correlations with age (r = 0.275, *p* < 0.01), height (r = 0.319, *p* < 0.01), weight (r = 0.319, *p* < 0.01), BMI (r = 0.241, *p* < 0.01), wrist circumference (r = 0.354, *p* < 0.01), PRA diameter (r = 0.521, *p* < 0.01), and UA diameter (r = 0.272, *p* < 0.01).

**Conclusion:**

The DRA diameter in children increases with age and size, making cardiac catheterization is theoretically feasible. Preoperative evaluation of the vessel diameter and intraoperative ultrasound-guided intervention are recommended for paediatric catheterization via the DRA access.

## Introduction

In recent years, proximal radial artery (PRA) access has emerged as an alternative access to femoral artery access for arterial catheterization in children, including cardiovascular interventions and invasive arterial blood pressure monitoring (1, 2). However, vessel intima injury during catheterization via PRA access and compression hemostasis can lead to radial artery occlusion (RAO) (3). RAO in children not only limits the future use of the radial artery but also affects limb development (4).

Compared to PRA access, distal radial artery (DRA) access in the anatomic snuffbox (AS) has been associated with a decreased risk of RAO, increased patient comfort and reduced hemostatic time (5–7). This approach has gained increased attention as an alternative for cardiac catheterization in adults (5–7). From the perspective of radial artery protection, catheterization via DRA access in children seems even more important (8). However, the DRA diameter in children is smaller than in adults, which increases the difficulty of puncturing and mastering the learning curve. Additionally, sheath-to-vessel mismatch may increase the risk of vascular injury (9).

To date, few studies have evaluated the DRA diameter and the feasibility of the catheterization via DRA access in children (10). Therefore, whether catheterization via DRA access in children is safe and effective needs further evaluation. This study aims to provide a reference for clinical catheterization via DRA access in children by measuring the DRA diameter in the AS.

## Methods

### Study design and population

We conducted a retrospective review of clinical and vascular ultrasound data of children (ages 3 to 12 years) who were scheduled for arterial blood gas analysis via the DRA due to serious respiratory diseases. These children were treated at the pediatric outpatient or inpatient department of the Changzhou Second People's Hospital between June 2023 and October 2023. The exclusion criteria included children aged ≤2 years, a history of PRA or DRA puncture, a history of peripheral artery disease, or a lack of ultrasound data. This study was approved by the Ethics Committee of Changzhou Second People's Hospital [Approval number: (2023)YLJSA069], and the requirement for informed consent was waived due to the retrospective nature of the study.

### Ultrasound examination

**Figure 1:**
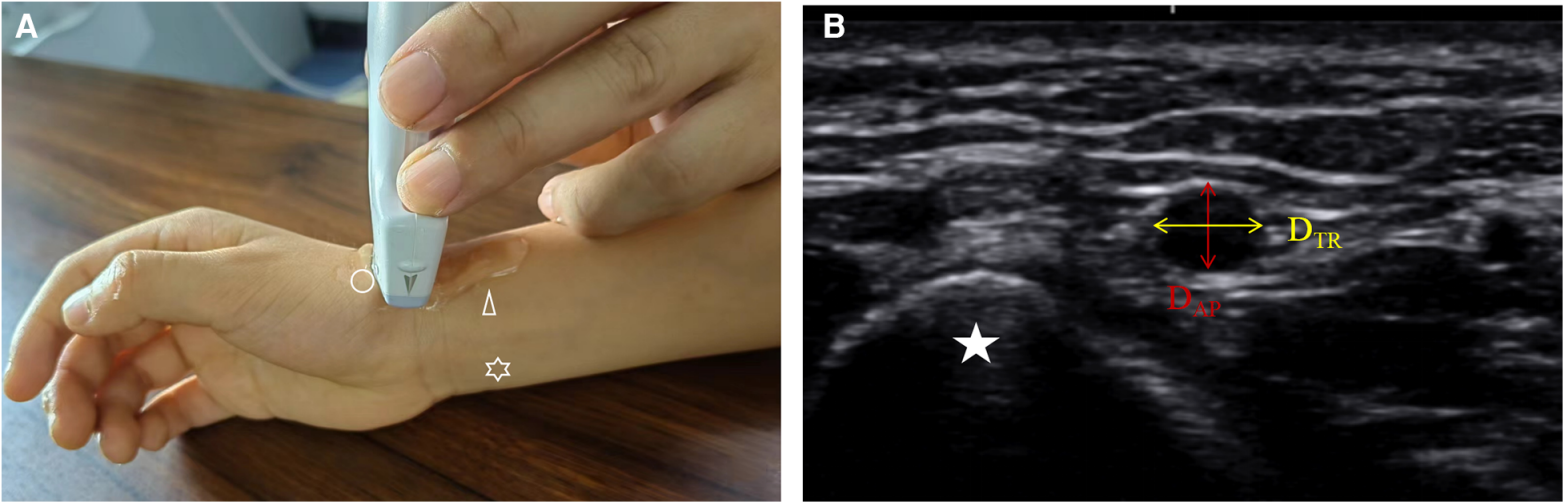
The measurement of DRA diameter in children by ultrasound. (**A**) the site of ultrasound measure. Circle: distal radial artery; Triangle: proximal radial artery; Star: ulnar artery. (**B**) the cross-sectional image of the distal radial artery in anatomic snuffbox. Star, scaphoid; D_TR_, transverse diameter; D_AP_, anteroposterior diameter.

A portable ultrasound machine (Konica Minolta SONIMAGE HS1 PLUS) with a “line array” probe (18-4 MHz) was used to measure the vessel diameter. During the examination, the operators gently placed the probe on the skin using their left thumb, index finger, and middle finger. Simultaneously, the little finger was positioned on the examinee's arm as a support to reduce the pressure of the probe on the tissue. Once the cross-sectional image of the vessel was displayed on the screen, slight adjustments were made to clearly observe the adventitia. The transverse diameter (D_TR_) and anteroposterior diameter (D_AP_) of the vessel were obtained simultaneously. To further reduce the risk of measurement error, the corrected diameter (D_C_) was calculated using the following formula: π(D_AP_/2) × (D_TR_/2) = π(D_C_/2)^2^ (11) (Figure 1).

The diameters of the PRA and ulnar artery (UA) were measured at the site 2 cm proximal to the wrist striation. The diameter of the DRA was measured at the AS. The depth of the DRA defined as the distance from the skin to the upper edge of the DRA. All diameters were measured three times by a single operator (Tao Chen), and the average value was taken. Additionally, 20 samples were measured simultaneously by two operators (Tao Chen and Yidong Zhao) to assess intra-observer agreement.

### Statistical analysis

Statistical analysis was carried out using SPSS 26.0 statistical software. Categorical variables are expressed as frequencies and percentages, and comparisons performed using the χ2 test. Continuous variables are expressed as the mean ± standard deviation, with comparisons conducted using two independent sample *t*-tests or one-way analysis of variance. The trend of the increase in DRA diameter with age was evaluated using the *P* for trend test. To analysis the proportions of DRA diameter >2.0 mm and >1.5 mm in different weight groups, the patients were divided into ten subgroups according to the declines of weight. Pearson correlation analysis was used to analyse the correlation between the DRA and other factors. The receiver operating characteristic curve (ROC) was used to analyze the predictive value of patients’ factors for DRA diameter >2.0 mm. Multiple comparisons were corrected by the Bonferroni method. Inter- and intraobserver agreement was assessed by calculating the intraclass correlation coefficients. A *p-*value <0.05 was considered statistically significant.

## Results

### Characteristics of the participants

A total of 412 children aged 3–12 years were included in the study. The characteristics of the participants are listed in Table 1. There were no significant differences in demographic characteristics, vital signs, or hand parameters between the male and female groups, except for wrist circumference. The proportion of males was 54.85% (226/412), and the mean BMI was 16.3 ± 3.17 kg/m^2^. Ultrasound data for 78.4% (323/412) of the individuals were obtained via the right hand. The depth of the DRA in males was significantly greater than in females (4.04 ± 1.06 mm *vs.* 3.91 ± 0.91 mm, *p *< 0.05).

**Table 1:** The characteristics of involved children.

	Total (*n* = 412)	Male (*n* = 226)	Female (*n* = 186)	*P*
Demographics				
Age, years	7.0 (5.0–9.0)	6.5 (4.0–9.0)	7.5 (5.0–9.0)	**0**.**019**
Height, m	1.28 ± 0.19	1.28 ± 0.19	1.29 ± 0.19	0.503
Weight, kg	28.16 ± 12.54	28.36 ± 13.04	27.92 ± 11.92	0.726
BMI, kg/m^2^	16.30 ± 3.17	16.52 ± 3.24	16.03 ± 3.07	0.121
Vital signs				
SBP, mmHg	101.21 ± 17.35	100.94 ± 18.35	101.55 ± 16.11	0.723
DBP, mmHg	65.19 ± 13.69	64.96 ± 13.92	65.47 ± 13.45	0.706
HR, beat/min	99.75 ± 14.91	100.51 ± 14.26	98.83 ± 15.66	0.256
Hand parameters				
Forearm length, cm	17.66 ± 3.68	17.46 ± 3.65	17.91 ± 3.70	0.221
Wrist circumference, cm	12.67 ± 1.66	12.86 ± 1.76	12.44 ± 1.51	0.012
Right side, *n* (%)	323 (78.4)	172 (76.1)	151 (81.2)	0.213

BMI, body mass index; DBP, diastolic blood pressure; HR, heart rate; SBP, systolic blood pressure; Bold values are significant *p*-values.

### DRA diameter in children

**Figure 2:**
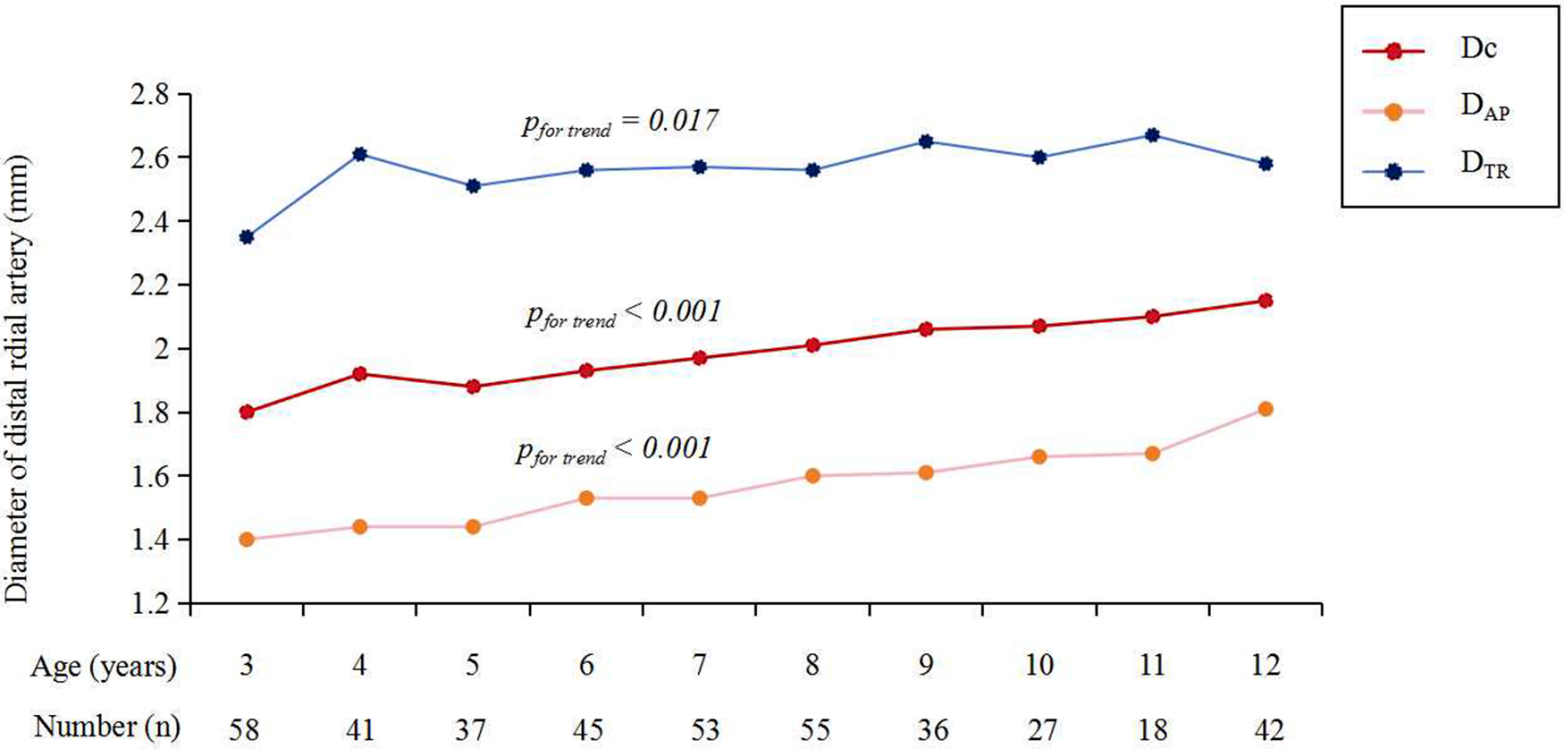
The diameter of DRA in different age groups. DRA, distal radial artery; D_AP_, anteroposterior diameter; D_c_, corrected diameter; D_TR_, transverse diameter.

The Dc of DRA in the AS was 1.97 ± 0.37 mm overall, with no significant difference between males (1.98 ± 0.38 mm) and females (1.95 ± 0.35 mm) (*p *= 0.457) (Table 2). The D_AP_, D_TR_, D_C_ of DRA increased significantly with age (Figure 2). The increasing trend of DRA diameter was also significant in both male and female subgroups.

**Table 2:** Vascular ultrasound data.

	Total (*n* = 412)	Male (*n* = 226)	Female (*n* = 186)	*P*
D_AP_ of DRA, mm	1.55 ± 0.32	1.56 ± 0.33	1.55 ± 0.30	0.817
D_TR_ of DRA, mm	2.54 ± 0.57	2.56 ± 0.58	2.51 ± 0.56	0.435
Dc of DRA, mm	1.97 ± 0.37	1.98 ± 0.38	1.95 ± 0.35	0.457
Dc of DRA >2.0 mm, *n* (%)	160 (38.8)	94 (41.6)	66 (35.5)	0.205
Dc of DRA >1.5 mm, *n* (%)	358 (86.9)	195 (86.3)	163 (87.6)	0.673
Dc of DRA >1.0 mm, *n* (%)	410 (99.5)	225 (99.6%)	185 (99.5)	1.000
PRA, mm	2.05 ± 0.33	2.08 ± 0.34	2.02 ± 0.32	0.052
UA, mm	1.88 ± 0.33	1.94 ± 0.34	1.81 ± 0.31	**<0**.**001**
Dc of DRA/CRA ≥1.0, *n* (%)	227 (55.10)	118 (52.21)	109 (58.60)	0.194

D_AP_, anteroposterior diameter; Dc, corrected diameter; DRA, distal radial artery; D_TR_, transverse diameter; PRA, proximal radial artery; UA, ulnar artery; Bold values are significant *p*-values.

**Figure 3:**
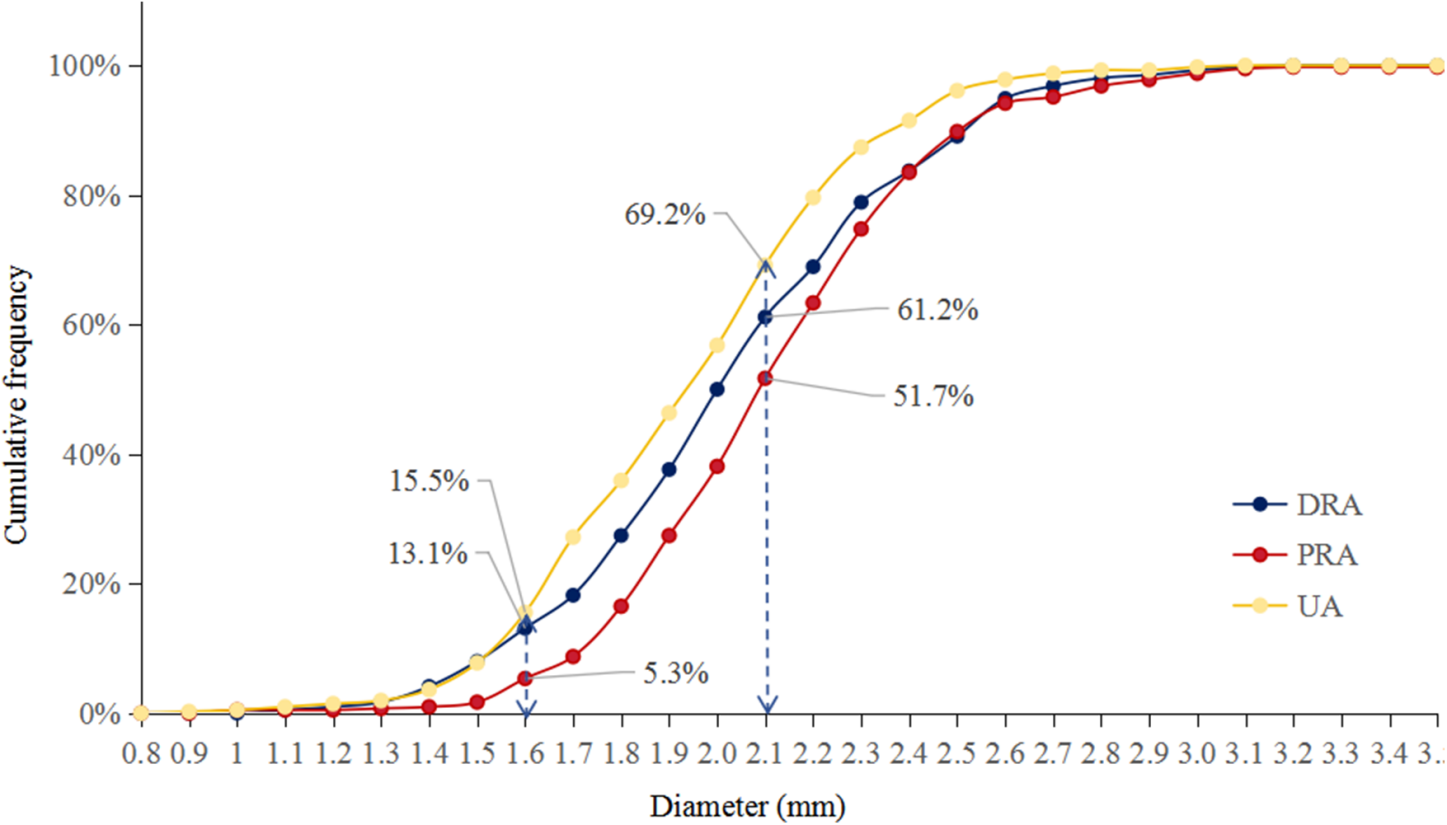
Cumulative frequency. The proportions of vessel diameter >2.0 mm was 38.8% in DRA, 48.3% in PRA, and 30.8% in UA, respectively. The proportions of vessel diameter >1.5 mm was 86.9% in DRA, 94.7% in PRA, and 84.5% in UA, respectively; DRA, distal radial artery; PRA, proximal radial artery; UA, ulnar artery.

**Figure 4:**
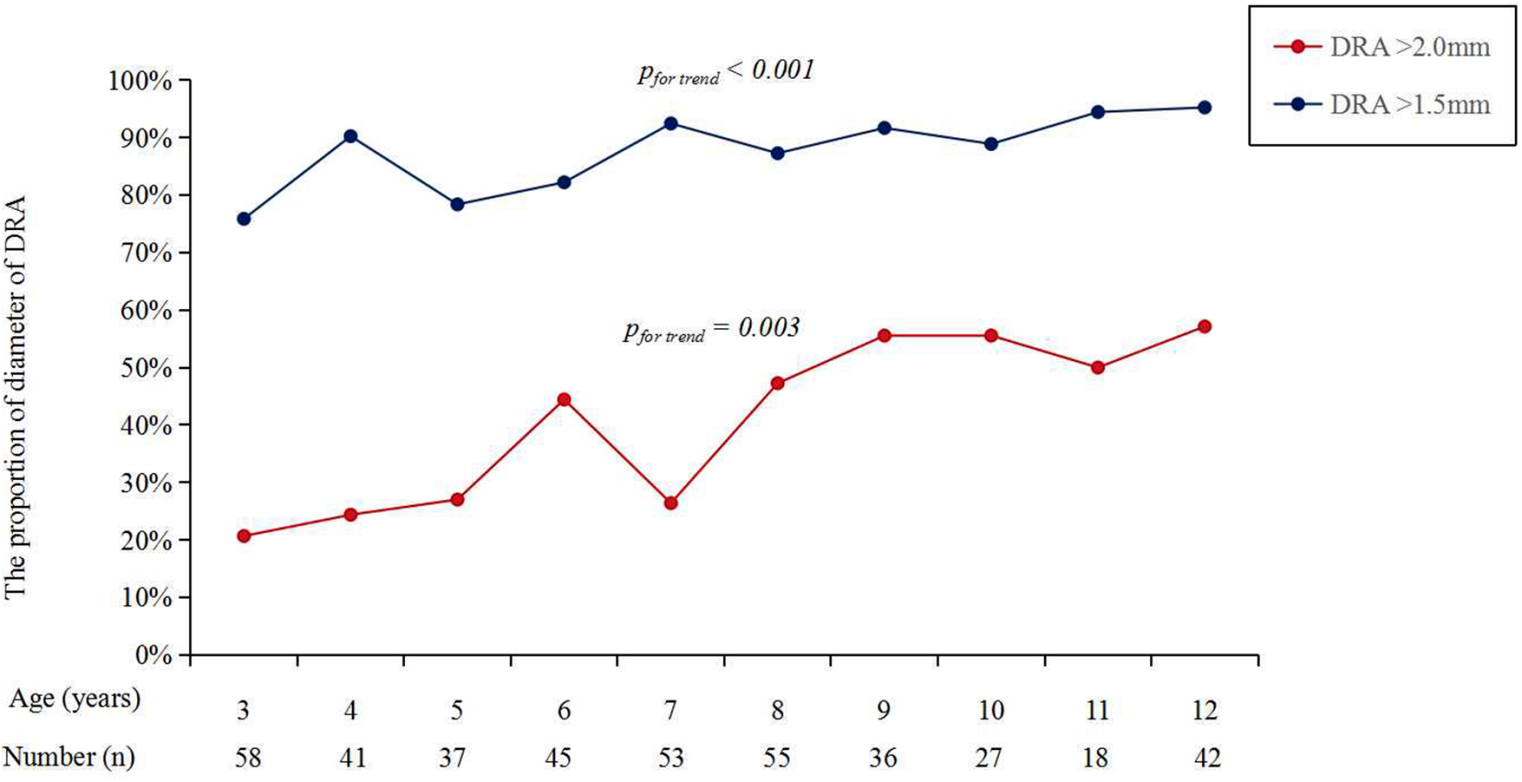
The proportions of DRA diameter over ages. DRA, distal radial artery.

**Figure 5:**
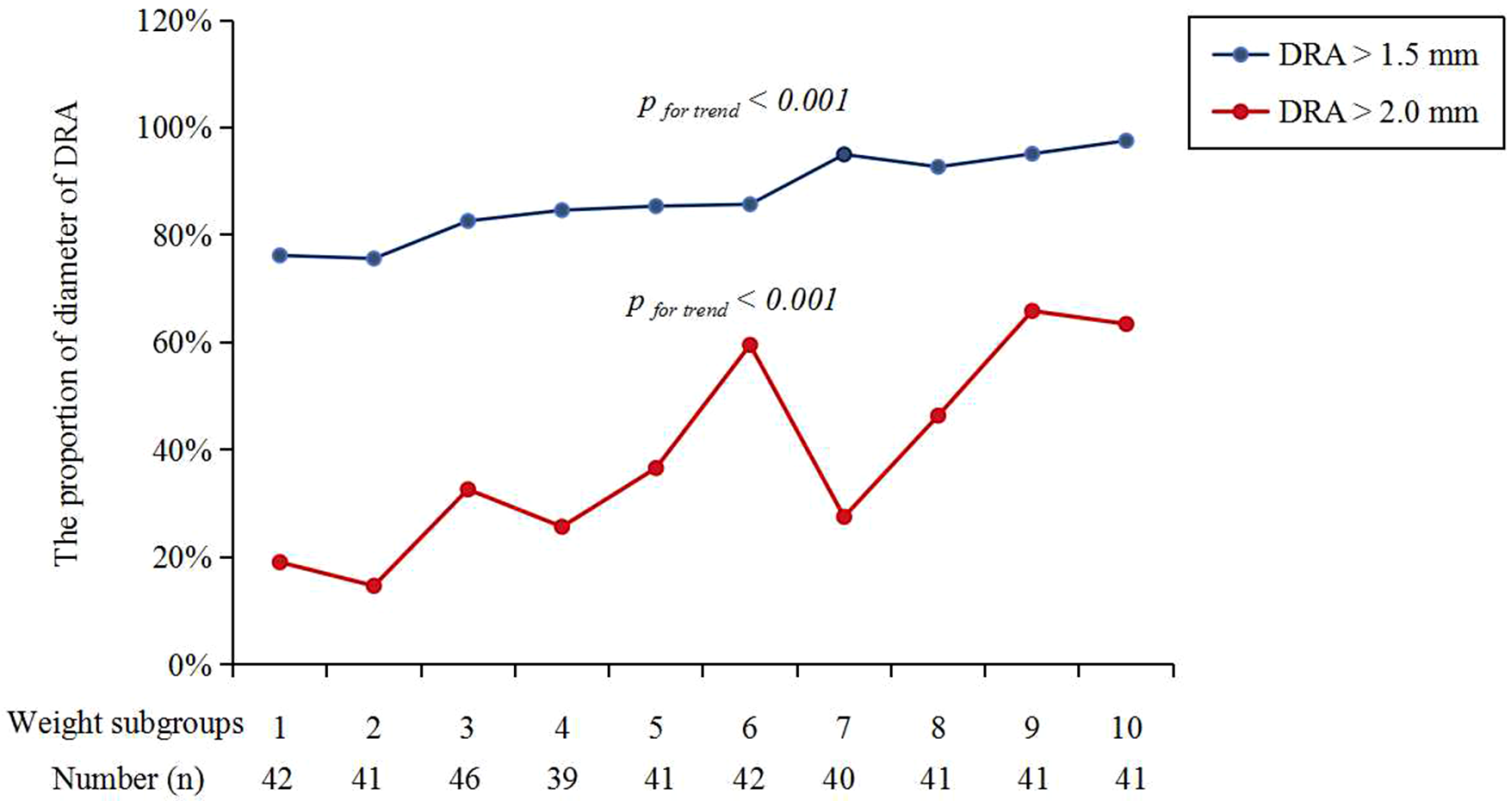
The proportions of DRA diameter over weight. DRA, distal radial artery.

Figure 3 shows the cumulative frequency of DRA diameter. Overall, the proportions of patients with a DRA diameter greater than 2.0 mm and 1.5 mm were 38.83% and 86.89%, respectively (Table 2). The proportions of patients with DRA diameters >2.0 mm and >1.5 mm according to age are shown in Figure 4, demonstrating a significant gradual increase with age (*p *< 0.01). Additionally, the proportions of patients with DRA diameters >2.0 mm and >1.5 mm according to weight are shown in Figure 5, demonstrating a significant gradual increase with weight (*p *< 0.001).

The DRA diameter was significantly smaller than the PRA diameter (1.97 ± 0.37 mm vs. 2.05 ± 0.33 mm, *p *< 0.001) but larger than the UA diameter (1.97 ± 0.37 mm vs. 1.88 ± 0.33 mm, *p *< 0.001) (Table 2). The percentages of individuals with a DRA/PRA ratio ≥1.0 were 55.10% overall, 52.12% in males, and 58.60% in females (Table 2). There was no significant difference in the proportion of patients with a DRA/PRA ratio ≥1.0 between male and female groups (*p *= 0.194).

### Correlations between the DRA diameter and other factors

The DRA diameter was significantly positively associated with age (r = 0.275, *p *< 0.01), height (r = 0.319, *p *< 0.01), weight (r = 0.319, *p *< 0.01), BMI (r = 0.241, *p *< 0.01), wrist circumference (r = 0.354, *p *< 0.01), PRA diameter (r = 0.521, *p *< 0.01), and UA diameter (r = 0.272, *p *< 0.01).

### Predictive factors for the DRA diameter >2.0 mm

ROC was used to analyze the predictive value of patients’ factors for DRA diameter >2.0 mm (Table 3). The AUC of age, height, weight, BMI and wrist circumference were 0.651, 0.676, 0.682, 0.632, and 0.689, respectively. The cut-off values were 7.5 years old, 1.335 m, 25.35 kg, 16.95 kg/m2, and 12.95 cm, respectively.

**Table 3:** Patients’ predictive factors for the DRA diameter >2.0 mm.

Index	Cut-off value	Sensitivity	Specificity	ROC	95%CI
Age, years	7.5	0.588	0.667	0.651	0.597–0.705
Height, m	1.335	0.556	0.71	0.676	0.623–0.729
Weight, kg	25.35	0.675	0.619	0.682	0.629–0.734
BMI, kg/m^2^	16.95	0.481	0.758	0.632	0.576–0.688
Wrist circumference, cm	12.95	0.588	0.734	0.689	0.636–0.742

BMI, body mass index; CI, confidence interval; DRA, distal radial artery; ROC, receiver operating characteristic curve.

## Discussion

To our knowledge, this was the first large sample-size study to measure the DRA diameter in children using high-frequency ultrasound. The study revealed that the Dc of the DRA was 1.97 ± 0.37 mm and that the DRA diameter gradually increased with age, height, weight, BMI, wrist circumference, PRA diameter, and UA diameter in children.

The majority of pediatric cardiocerebrovascular interventions can be successfully performed using femoral artery access. In recent years, PRA, carotid, and axillary arterial access have emerged as attractive alternatives to femoral access and have been reported as safe and effective for cardiocerebrovascular intervention (12–14). The 4 Fr and/or 5 Fr sheath can be safely and effectively used for cardiovascular diagnosis and intervention via PRA access in children with diameters of 1.96 mm and 2.29 mm respectively (10). In children, the mean PRA diameter was reported to be 1.39 mm in females and 1.57 mm in males, correlated with sex, age, and body weight (15). Alehaideb A et al. reported that the mean corrected PRA diameter was 1.86 mm in 134 children younger than 18 years. However, studies have shown that cardiovascular intervention via PRA access may cause injury to the forearm radial artery in adults, increase the thickness of the vessel intima, and raise the incidence of RAO, potentially limiting the reuse of the radial artery (16).

The DRA access has been proven to have significantly fewer access-related complications, including forearm RAO, due to its unique anatomical structure (7). Moreover, the DRA has safely and effectively used as an alternative access for invasive arterial blood pressure monitoring under surgical anesthesia and in the ICU due to its good blood pressure consistency with the PRA (17, 18). In adults, the DRA diameter was reported to be about 2.0 mm, smaller than the PRA diameter, with a DRA/PRA ratio of approximately 0.8 (19–22). However, the DRA is not always smaller than the PRA (19). Predictive factors for DRA diameter vary; for instance, in Koreans, female sex and a low BMI were reported as independent predictors of a DRA diameter <2.3 mm (22), while in Japanese patients, no association was found between patient characteristics and vessel diameter (20).

Few studies have evaluated the DRA diameter and explored the feasibility of interventional diagnosis and treatment via DRA access in children (10). Theoretically, DRA access may provide better protection for the forearm radial artery in children compared to PRA access. Although DRA puncture in children is more challenging due to the small vessel size. methods such as the use of thin-walled sheaths, vasodilators, and ultrasound-guided puncture can significantly increase the success rate of small vessel punctures and reduce puncture-related complications (23–25). In a small sample size study, Srinivasan VM et al. reported that the DRA diameter was 2.09 ± 0.54 mm, similar to the PRA diameter (10). In our study, the corrected DRA diameter in the AS was 1.97 ± 0.37 mm and gradually increased with age. It was significantly smaller than the PRA and larger than the UA. More than 50% of children had a DRA/PRA ratio ≥1.0, significantly higher than in adults (19). This discrepancy may be due to developmental imbalances in children's vessels and superficial location of the DRA in the AS. Despite using appropriate methods to relieve probe pressure during examination, the corrected diameter may not reflect the true lumen size. Compared to adults, children have fewer underlying diseases, which might affect vessel size differently. In this study, the DRA diameter was significantly positively associated with age, height, weight, BMI, wrist circumference, PRA diameter, and UA diameter.

### Limitations

First, the children in the study were not consecutively enrolled and were from a single hospital, which may have led to the selection bias. Second, although the sample size was relatively large, the number of patients per age group was small. Previous studies have shown that the increase in the diameter of the PRA plateaus after 12 years of age (11). In this study, the diameter of the DRA gradually increased with age until 12 years of age; however, we cannot conclude whether it has reached a plateau. Third, the diameter of the DRA was measured from adventitia to adventitia, and corrected using a formula, which does not reflect the true inner diameter of the DRA. Fourth, there was a lack of comparison between the two sides. Fifth, the children in this study were from urban areas in Changzhou, Jiangsu, with relatively higher economic status compared to children from other regions. Differences in growth and development among children might affect the size of the DRA.

## Conclusion

The diameter of the DRA in children increases with age, making catheterization via the DRA access theoretically feasible. Preoperative evaluation of the vessel diameter and intraoperative ultrasound-guided intervention are recommended for pediatric catheterization via DRA access.

## Data Availability

The raw data supporting the conclusions of this article will be made available by the authors, without undue reservation.
